# Cardiovascular risk outcome and program evaluation of a cluster randomised controlled trial of a community-based, lay peer led program for people with diabetes

**DOI:** 10.1186/s12889-016-3538-3

**Published:** 2016-08-24

**Authors:** M. A. Riddell, J. A. Dunbar, P. Absetz, R. Wolfe, H. Li, M. Brand, Z. Aziz, B. Oldenburg, the Australasian Peers for Progress Diabetes Project Investigators, Brian Oldenburg, James A. Dunbar, Prasuna Reddy, Virginia Hagger, Greg Johnson, Maximilian de Courten, Rory Wolfe, Rob Carter, Pilvikki Absetz, Anuar Zaini

**Affiliations:** 1 Department of Epidemiology and Preventive Medicine, Faculty of Medicine Nursing and Health Science, Monash University, The Alfred Centre, 99 Commercial Road, Melbourne, Victoria 3004 Australia; 2 Deakin Population Health Strategic Research Centre, Deakin University, Melbourne, Australia; 3 School of Health Sciences, University of Tampere, Tampere, Finland; 4 Institute of Chronic Disease Control, Beijing Centers for Disease Control and Prevention, Beijing, People’s Republic of China; 5 Melbourne School of Population and Global Health, University of Melbourne, Melbourne, Australia

**Keywords:** Diabetes, Self-management, Peer support, Medication adherence, Diabetes self-care behaviours, UKPDS

## Abstract

**Background:**

The 2013 Global Burden of Disease Study demonstrated the increasing burden of diabetes and the challenge it poses to the health systems of all countries. The chronic and complex nature of diabetes requires active self-management by patients in addition to clinical management in order to achieve optimal glycaemic control and appropriate use of available clinical services. This study is an evaluation of a “real world” peer support program aimed at improving the control and management of type 2 diabetes (T2DM) in Australia.

**Methods:**

The trial used a randomised cluster design with a peer support intervention and routine care control arms and 12-month follow up. Participants in both arms received a standardised session of self-management education at baseline. The intervention program comprised monthly community-based group meetings over 12 months led by trained peer supporters and active encouragement to use primary health care and other community resources and supports related to diabetes. Clinical, behavioural and other measures were collected at baseline, 6 and 12 months. The primary outcome was the predicted 5 year cardiovascular disease risk using the United Kingdom Prospective Diabetes Study (UKPDS) Risk Equation at 12 months. Secondary outcomes included clinical measures, quality of life, measures of support, psychosocial functioning and lifestyle measures.

**Results:**

Eleven of 12 planned groups were successfully implemented in the intervention arm. Both the usual care and the intervention arms demonstrated a small reduction in 5 year UKPDS risk and the mean values for biochemical and anthropometric outcomes were close to target at 12 months. There were some small positive changes in self-management behaviours.

**Conclusions:**

The positive changes in self-management behaviours among intervention participants were not sufficient to reduce cardiovascular risk, possibly because approximately half of the study participants already had quite well controlled T2DM at baseline. Future research needs to address how to enhance community based programs so that they reach and benefit those most in need of resources and supports to improve metabolic control and associated clinical outcomes.

**Trial registration:**

Australian New Zealand Clinical Trials Registry (ANZCTR) ACTRN12609000469213. Registered 16 June 2009.

**Electronic supplementary material:**

The online version of this article (doi:10.1186/s12889-016-3538-3) contains supplementary material, which is available to authorized users.

## Background

Recent estimates from the 2013 Global Burden of Disease Study demonstrate the continued increasing health burden of diabetes and the challenge it poses for the health systems of all countries [1]. Cardiovascular disease (CVD) is a leading cause of death associated with type 2 diabetes (T2DM) with more than double the risk of CVD-related mortality, compared to those with normal blood glucose levels [2]. In Australia in 2011, for those cases in which diabetes was listed as the underlying cause of death, chronic heart disease (CHD) was associated in 64 % of deaths, and where CHD was listed as the underlying cause of death, diabetes was associated in approximately 24 % of deaths [3]. The United Kingdom Prospective Diabetes Study (UKPDS) indicated that optimal glycaemic and blood pressure control were associated with a reduced risk of complications [4, 5]. Therefore programs which support the adoption of self-management behaviours to improve glycaemic control, reduce blood pressure and other clinical measures to reduce the risk of complications are required.

The chronic and complex nature of T2DM requires active self-management by the person with the condition in order to achieve optimal glycaemic control, appropriate use of available clinical services, and other resources and supports. This requires adoption of self-care behaviours and key lifestyle behaviours to promote optimal glycaemic and blood pressure control, smoking cessation, diet, regular physical activity, medication adherence and monitoring of blood glucose levels (BGL).

Peer support delivered via community based programs may assist people with diabetes to improve their glycaemic control [6–9]. Indeed, Fisher et al. propose that peer support can assist with daily diabetes management, improve linkages with clinical care providers and provide ongoing emotional and social support [10]. Several studies, published subsequent to the conduct of this study, have reported the provision of peer support to people with diabetes in a variety of clinical and community settings and in different countries [7, 8, 11–18]. Several of these have looked at glycated haemoglobin (HbA1c) as the outcome and a number have reported a reduction in HbA1c [7, 8, 18–20]. It is likely that in one of these studies the improvement resulted from insulin initiation [21], and in another, the reduction was only evident in a selected population [19]. A further two studies examined the effects of group-based support, but found no significant improvements in HbA1c [15, 16], however group based peer support, as delivered by Simmons et al., resulted in significant reduction of systolic blood pressure associated with a relative reduction in myocardial infarction of 2 – 4 % and stroke of 4–6 % [15]. Furthermore, the added value of peer support for patients already receiving or having access to adequate clinical care may not lead to additional clinical benefits [22]. A secondary analysis of the study by Thom et al. [8] revealed that those participants with poorer glycaemic control benefitted more from peer support compared to those participants with better glycaemic control [23].

Other studies have examined peer support delivery mechanisms, for example, in one study, telephone-based peer support did not improve the cardiovascular outcomes over and above integrated care delivered by using a web-based quality improvement program, although it did reduce both distress and hospitalizations among those initially more distressed [11]. Another study demonstrated reductions in HbA1c for those receiving peer led group based peer support and those receiving support via telephone-based outreach program delivered by community health workers after a 6 month diabetes self-management education (DSME), however, sustained benefits were only apparent in the group based peer led support recipients [17]. A recent meta-analysis of 13 randomised control trials of peer support to improve glycaemic control in patients with T2DM concluded that receipt of peer support significantly contributed to a reduction in HbA1c [24]. Furthermore, trials in which contact between peers and supporters were moderate or higher in frequency showed a greater and more significant effect size compared to programs in which the contact frequency was low.

Although there is now some good evidence that peer support can contribute to improving the clinical outcomes for those with diabetes and other chronic conditions [9, 25], several studies highlight that peer support may not achieve clinical benefits for those already receiving high quality clinical care [11, 22] or those already in moderately good control [15]. Furthermore, it is not very clear how variations in contextual and other factors might also impact on implementation and thereby, clinical and other health outcomes. Such factors might include the target population, health care systems and/or differences in community settings and contexts, intervention design (e.g., contents and components) and the intensity and fidelity of program delivery (e.g., peer supporter training and competence in delivery). There is an important need for research to understand more about the importance of and/or interplay among these different factors.

In 2008, the Peers for Progress, a program conducted under the auspices of the American Academy of Family Physicians Foundation, supported eight studies to investigate and expand the evidence base for peer support using different approaches to program design and delivery in USA, Australia, Hong Kong and United Kingdom. All programs were encouraged to pursue standardisation by function rather than content [6, 26, 27], with the agreed upon core functions, being: 1) assistance in daily management, that is, assisting with the” how” of daily self-management); 2) providing social and emotional support to individuals; 3) promoting and supporting regular linkage to clinical care; and finally, 4) provision of ongoing and sustained support to assist with the lifelong needs of diabetes self-care management.

The Australasian Peers for Progress (PfP) Project was undertaken in collaboration with Diabetes Australia-Vic (DA-Vic), the primary diabetes NGO in the Australian state Victoria. DA-Vic already had a pre-existing peer support program which primarily focused on bringing people with diabetes together to share their knowledge and experiences. However, the Peers for Progress program was designed to provide more structured support and assistance to the participants in the groups by focusing on key self-management behaviours and goals and by providing structured training and support to the peer supporters to deliver this kind of program. Therefore, we aimed to evaluate if a more structured peer support program would result in significant clinical and self-management outcomes. Following the earlier publication of the study protocol [28], this paper now reports on the 12-month outcomes of the program on the control and self-management of T2DM among program participants, as well as some key findings from the evaluation of program implementation.

## Methods

### Study design and setting

The published study protocol for this cluster randomised controlled trial of a group-based peer support program was implemented and evaluated in accordance with the requirements of the Consolidated Standards of Reporting Trials (CONSORT) statement and its extension to cluster randomised trials [28, 29]. Trial participants were recruited from a national diabetes registry of more than one million Australians with diabetes, the Australian National Diabetes Services Scheme (NDSS) [30]. Twenty-four geographic communities within the state of Victoria in Australia (Local Government Authorities (LGAs)) were selected, from which study participants were recruited. The project received ethics approval from the Monash University Human Research Ethics committee (MUHREC) Project number CF09/1692 – 2009000920.

### Recruitment and allocation

We approached participants from Australia’s NDSS Registry if they had been registered for more than 12 months, resided in one of the selected LGAs and were aged between 25 – 75 years. Interested recipients were contacted by a member of the project team. Exclusion criteria included any current medical or related condition likely to prevent study participation over a 12 month period and/or poor comprehension in English. We aimed to recruit an average of 10 – 15 participants and two peer supporters with diabetes from each of the 24 study locations (“clusters”). For each of three of the eight state health regions equal numbers of groups (*n* = 4) were randomly allocated to receive the intervention and usual care. In summary, 24 groups (clusters) were allocated to either intervention or usual care. All participants were provided with the anthropometry and pathology results of their baseline, 6 and 12 month testing, and an accompanying letter encouraging them to share the results with their clinical team.

### Diabetes self-management education

Prior to randomisation, all enrolled participants were invited to attend a 7-hour diabetes self-management education (DSME) course that was delivered by credentialed diabetes educators and dieticians from DA-Vic. The DSME program presented basic disease information about diabetes and its clinical management, the key component being self-management practices including diet and physical activity, prevention and management of disease complications, and medications. The participants in this program received a comprehensive manual. The purpose of this manual was to supplement and reinforce information received during the DSME session. For those who could not attend the face-to-face course, an education manual and an educational DVD, filmed during one of the program education sessions and supplemented with visual material, was provided to these participants. Participants were asked to complete a brief evaluation form at the end of the education session rating their general satisfaction with the session.

### Intervention program

The intervention program comprised four inter-related components that have been previously described in detail [28]: 1) assistance with the” how” of daily self-management; 2) provision of social and emotional support; 3) promotion and support of regular linkage to clinical care; and 4) provision of ongoing and sustained support to assist with the lifelong needs of diabetes self-management. Volunteer peer supporters led a monthly community-based group meeting for participants, and contact between supporters and participants outside these sessions was also encouraged and facilitated. Meetings were scheduled and organised by the supporters in consultation with group members. Prior to each meeting, participants received a brief phone call from the peer supporter reminding them of the upcoming meeting and seeking their intention to attend. Meetings were scheduled for 90 minutes and meeting content and delivery was based on the guidelines, provided in the peer supporter handbook, with these being delivered in consultation with group members. Each program session provided a list of suggested meeting topics and issues which focused on the key diabetes self-management behaviours, ways to implement socio-behavioural strategies such as goal setting and monitoring into daily life as well as to improve participants’ “links” with clinical care. A regular review of their clinical targets - and also the utilization of other appropriate community resources and supports were also incorporated into the meetings. Groups were especially encouraged to; 1) use all of the available information resources; 2) arrange visits to their group meetings by local health care professionals such as dietitians and diabetes educators; and 3) participate in shared activities with each other outside of the group meetings such as walks, tai-chi or other exercise classes as well as social gatherings. A bi-monthly newsletter from the project team to group members contained project news, group profiles and photos, recipes and other information to supplement the group meetings.

For delivery of the program, the peer supporters received two and a half days of training from a credentialed diabetes nurse educator experienced in group facilitation and effective communication as previously described [28]. Briefly peer supporter training aimed to equip supporters with communication and group facilitation skills. This training aimed to help them support their group members to tell their stories, set goals, problem solve, increase awareness and linkages with the available health system, optimize self-management behaviours, including glucose monitoring, dietary changes and physical activity, as well as provide emotional support. Peer supporters were also supported by the project team through weekly teleconferences, to which they were asked to attend at least once per month. Follow-up notes and actions were provided to all supporters via a weekly email.

#### Usual care

Apart from individualised feedback on clinical measures collected as part of the baseline, 6 and 12 month assessments and participation in the diabetes education session, participants in usual care were offered no other formal support for the duration of the research trial.

### Measurement and evaluation

Participants underwent anthropometric measurements by trained research support staff according to the WHO STEPwise approach to Surveillance (STEPS) protocol [31] with some minor modifications as previously described [28]. Additionally participants completed a self-administered survey at baseline, 6 and 12 months after the start of the intervention seeking information on demographic, clinical, behavioural, quality of life (EQ-5D [32], diabetes distress (DDS-4) [33]), depression (PHQ-9 [34]) diabetes self-care [35], satisfaction with diabetes support [36] and diabetes services utilisation (GPAQ ver 2.1 [37]) as previously described [28]. Participants were provided with a pathology request form to obtain blood tests at a local laboratory including HbA1c, total Cholesterol (TC), high-density lipoprotein (HDL), low-density lipoprotein (LDL), TC/HDL ratio, triglycerides at each time point. Results were returned to the project team; they were then provided to the participant with a letter suggesting they also share the results with their medical practitioner. Laboratories were accredited by National Association of Testing Authorities (http://www.nata.com.au/nata/) and all methods for determining HbA1c% were National Glycohemoglobin Standardization Program (NGSP) approved (http://www.ngsp.org/). HbA1c % measurements have been converted to the new International Federation of Clinical Chemistry (IFCC) unit (mmol/mol) [38] and are presented in parentheses for this publication.

### Data analysis

The primary outcome measure was cardiovascular disease risk at 12 months using the United Kingdom Prospective Diabetes Study (UKPDS) 5 yr risk calculation [39]. A sample size of 100 participants in each arm of the study at 12 months follow up was calculated as being sufficient to detect a mean change of −2.0 % in the peer support groups compared to a mean change of 0 % in the usual care group with 80 % power [28, 40]. Both groups aged by 1 year which increased their UKPDS risk score and we anticipated this to be offset by improvements in their systolic blood pressure (SBP) and total cholesterol/HDL cholesterol ratio, and possibly their HbA1C and smoking status.

The clustering of individuals by peer group arm or usual care arm did not require any inflation of the sample size because the comparisons across clusters were within-person changes over time and these were assumed to have negligible intra-cluster correlation. To allow for attrition we aimed to recruit 12 groups and 120 participants per arm at baseline.

Baseline demographic characteristics of the study population were presented as means, standard deviations (SD), frequencies and percentages based on the different variables’ type: continuous, binary or categorical. Independent samples *t*-test, chi-square test and Wilcox rank-sum test were used to check the differences of baseline characteristics between the intervention group and control group. Linear regression with cluster-based standard errors was used to compare the differences of continuous variables, including the UKPDS 5 yr risk, between usual care and intervention at 12 months with adjustment for baseline. Multilevel ordered logistic regression was used to compare the changes of Patient Health Questionnaire-9 (PHQ-9) [41] and Morisky Medication Adherence Scale-8 (MMAS-8) [42] between usual care and intervention from baseline to 12 months. Logistic regression with cluster-based standard errors was used to compare the differences of dichotomised HbA1c between usual care and intervention at 12 months with adjustment for baseline. To examine sensitivity of primary outcome results to missing data the analysis of 5 yr UKPDS risk was also conducted using multiple imputations. All analyses were completed in the Stata statistical software package (version 13.0).

## Results

### Study recruitment and response rates

**Fig. 1:**
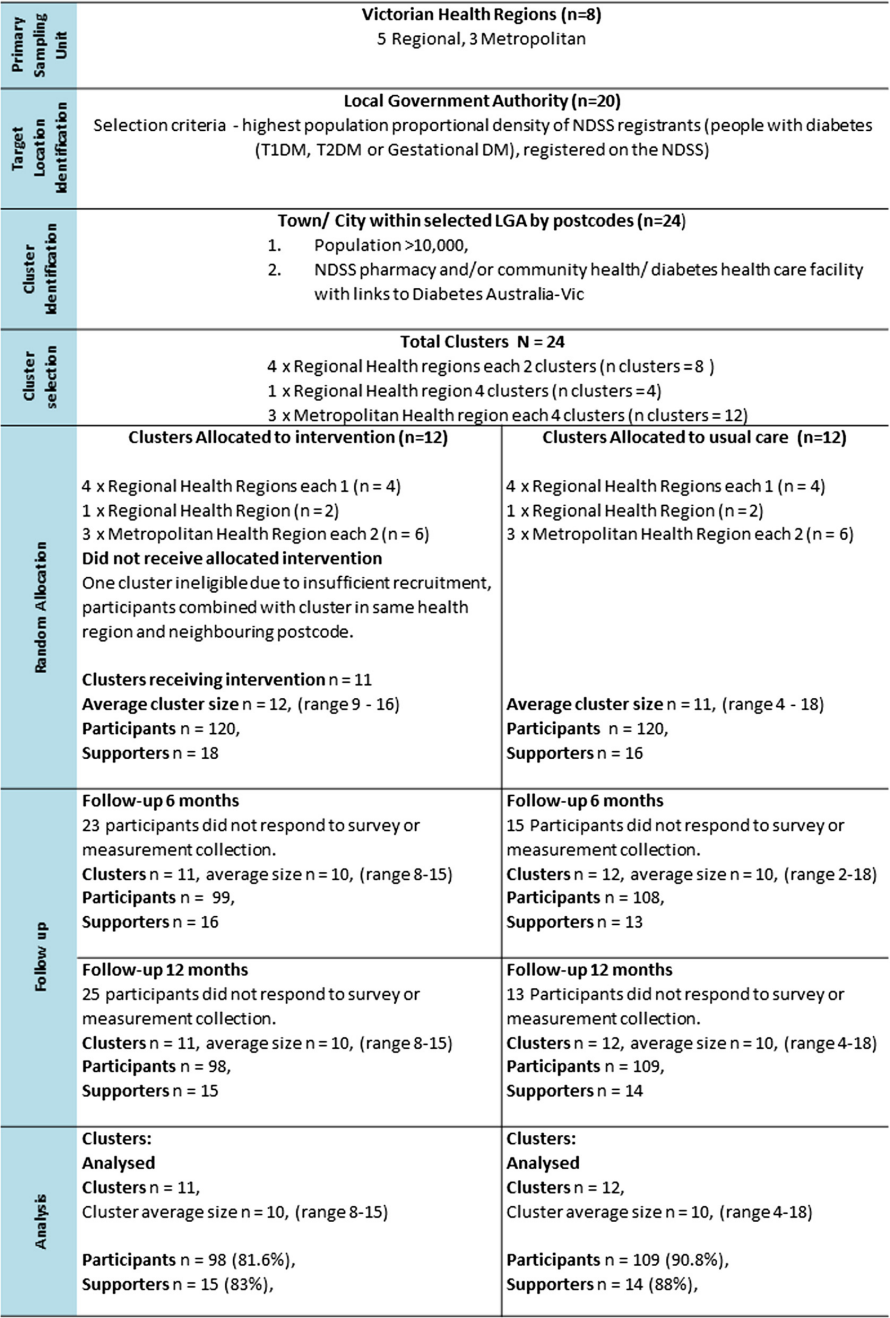
CONSORT diagram (recruitment & participant flow)

**Fig. 2:**
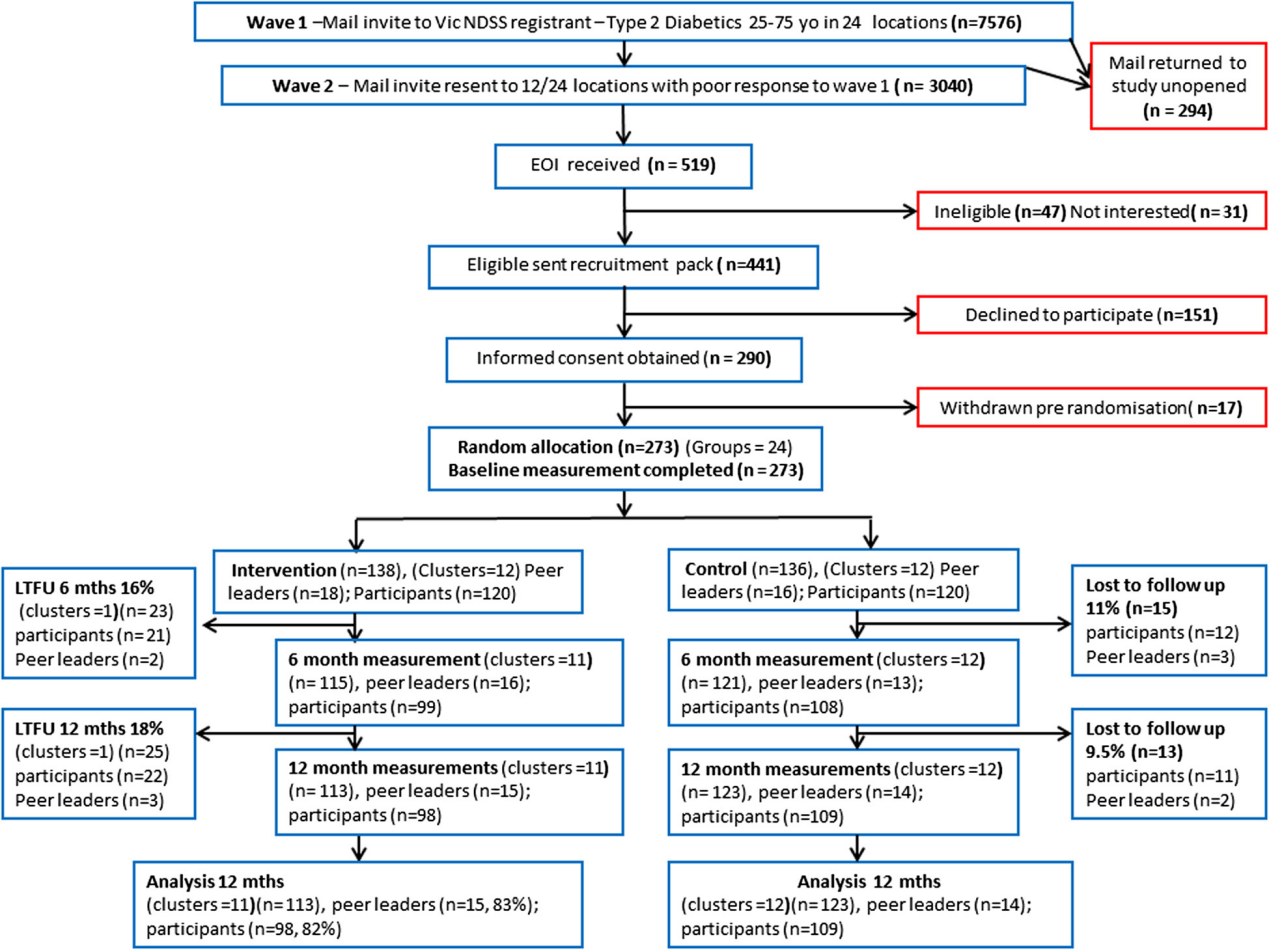
Individual participant flow

Figure 1 CONSORT diagram shows the sampling strategy and recruitment of clusters for the study. Figure 2 describes the participant flow throughout the 12 month intervention. Recruitment of participants occurred from May to July 2010. From 24 locations in regional and metropolitan Victoria, 7576 people registered with the NDSS with T2DM were invited by mail to participate in the study. Follow up letters were sent to 3040 of the registrants from locations in which initial response was poor. Incorrect address details or deceased addressee resulted in the return of 294 invitation letters. Expressions of interest to participate in the study were received from 501 persons (response rate = 6.9 % (501/7282)). Of these, 441 persons were eligible to enter the study and 151 of these people declined to participate after receiving further study requirement details. Written informed consent was obtained from 290 persons (65.7 % (290/441) of eligible respondents). Seventeen participants withdrew prior to the start of the intervention and thus, 273 participants were invited to attend local or centrally located diabetes self-management education sessions during August to October 2010. During this time baseline measures were collected from all participants. Of these 273 participants, 33 volunteered and were deemed suitable as peer supporters resulting in 240 peer group support participants. Random allocation of location clusters was undertaken in October 2010, following baseline measurement and conduct of the diabetes self-management education sessions. Training of the intervention peer supporters took place following these steps and peer group meetings were initiated in November 2010.

All intervention participants who attended the group meetings were additionally invited to participate in the process evaluation. A survey questionnaire was used at the end of 6 and 12 months to assess: participation and retention in the intervention; implementation fidelity; group effectiveness; participants’ satisfaction with intervention materials; participants’ willingness to continue to use the strategies learnt through this intervention; and to assess the extent of support provided by peers and by the research team as perceived by participants. The results of the detailed process evaluation measures will be reported separately (Aziz et al., under preparation).

### Clinical and anthropometric outcomes

Table 1 shows baseline characteristics of study participants to be generally balanced by intervention and usual care allocation. The median duration of diabetes was 2 years longer in intervention participants compared with usual care participants (9 yr. vs 7 yr., *p* = 0.01). At baseline the mean UKPDS for male participants was 11.5 % (SD 7.5 %) and for females 4.2 % (SD 2.8 %). There was higher baseline risk in the intervention arm. However, risk scores in both study arms reduced by similar amounts over 12 months with the difference between arms in risk changes being zero (95 % CI −0.011, 0.011, *p* = 1.00; Table 2). Results of this analysis based on multiple imputations had similar results. There were no significant differences in other clinical outcomes (Table 3).

**Table 1:** Baseline characteristics (participants)

	Control(*n* = 120)		Intervention(*n* = 120)		Total	
	*n*	Result	*n*	Result	*n*	Result
Age (mean ± SD)	115	60.5 ± 8.7	116	61.3 ± 9.3	231	60.9 ± 9.0
Sex (Male, %)	120	62 (51.7)	120	60 (50.0)	240	122 (50.8)
Metro/rural (metro, %)	120	61 (50.8)	120	59 (49.2)	240	120 (50.0)
Country of birth (Aust., %)	113	72 (63.7)	113	66 (58.4)	226	138 (61.1)
Language (English, %)	113	104 (92.0)	113	97 (85.8)	226	201 (88.9)
Ethnicity (count, %)	112		110		222	
Caucasian		98 (87.5)		92 (83.6)		190 (85.6)
South East Asian		6 (5.4)		9 (8.2)		15 (6.8)
Indian sub-continent		4 (3.6)		7 (6.4)		11 (5.0)
Other		4 (3.6)		2 (1.8)		6 (2.7)
Living arrangement (count, %)	115		116		231	
Single/no dependents		31 (27.0)		25 (21.6)		56 (24.2)
Single/with dependents		9 (7.8)		10 (8.6)		19 (8.2)
Married/no dependents		44 (38.3)		48 (41.4)		92 (39.8)
Married/with dependents		29 (25.2)		28 (24.1)		57 (24.7)
other		2 (1.7)		5 (4.3)		7 (3.0)
Employment (working, %)	113	50 (44.3)	113	41 (36.3)	226	91 (40.3)
Smoking status (count, %)	109		110		219	
Current		6 (5.5)		12 (10.9)		18 (8.2)
Previous		42 (38.5)		43 (39.1)		85 (38.8)
Never		61 (56.0)		55 (50.0)		116 (53.0)
Median age at diagnosis	54 yr. (IQR: 46–60)		54 yr. (IQR: 46–60)		54 yr. (IQR:46–40)	
Median duration of diabetes	7 yr. (IQR 3.3-10)		9 yr. (IQR: 5–12)		8 yr. (IQR: 4.5–10)

**Table 2:** Five year Cardiovascular Disease Risk (UKPDS risk score) at baseline and 12 months for intervention and control groups

Participants	Usual care (*n* = 120)	Intervention (*n* = 120)
UKPDS risk Baseline, mean (SD)	0.075 (0.06)	0.084 (0.07)
UKPDS risk 12 months, mean (SD)	0.070 (0.06)	0.079 (0.07)
Change in UKPDS risk 12 months – baseline, mean change	−0.005 (*p* = 0.18)	−0.005 (*p* = 0.31)
Difference in UKPDS risk changes	−0.000 (95 % CI −0.011, 0.011, *p* = 1.00)

**Table 3:** Mean biochemical and anthropometric measures at baseline and 12 months

		Usual Care				Intervention				
	Target	Baseline Mean ± SD (range)	12 months Mean ± SD (range)	Mean change	95 % CI	Baseline Mean ± SD (range)	12 months Mean ± SD (range)	Mean change	95 % CI	*p* value^*^
HbA1c (%)	≤7.0 %	7.2 ± 1.3 (5–13.8)	7.3 ± 1.2 (5.1 – 12.1)	0.22	(0.02, 0.41)	7.3 ± 1.0 (5.5 – 10.3)	7.3 ± 1.1 (5.5 – 11.3)	0.06	(-0.13, 0.24)	0.53
Weight (kg)		88.0 ± 20.5 (45.4 – 169.4)	87.7 ± 19.8 (41.5 – 132.6)	−0.52	(-1.50, 0.42)	88.9 ± 17.8 (51.3 – 137.3)	87.3 ± 18.4 (52.5 – 136.9)	−0.30	(-1.06, 0.46)	0.71
BMI	≤25	31.9 ± 6.8 (17.6 - 54.1)	31.9 ± 6.7 (21.1 – 49.7)	−0.11	( −0.46, 0.24)	31.7 ± 5.9 (18.9 - 54.9)	31.5 ± 6.2 (19.7 – 54.0)	−0.10	(−0.38, 0.17)	1.0
Waist Circum. (cm)	Men <94 Women <80	106.8 ± 16.7 (64 – 153.5)	106.9 ± 16.0 (69.5 – 138)	0.9	(−0.5, 2.3)	106.9 ± 14.6 (73.5 – 145)	106.8 ± 15.7 (78.5 - 182)	1.1	(−1.2, 3.4)	0.99
SBP (mmHg)	≤130	137.4 ± 16.8 (95 – 192.7)	128.1 ± 17.1 (96–189)	−9.1	(−12.8, −5.4)	134.9 ± 15.5 (95 – 170)	130.9 ± 16.3 (98–196.7)	−5.1	(−9.1, −1.0)	0.19
DBP (mmHg)	≤80	81.4 ± 11.1 (55 – 111)	75.3 ± 11.3 (53–116.7)	−6.3	(−9.0, −3.6)	79.3 ± 10.4 (50.7 – 106.7)	74.6 ± 11.0 (47 – 110)	−4.0	(−6.6, −1.4)	0.82
Total Cholesterol	<4.0	4.5 ± 1.0 (2.6 - 7.6)	4.3 ± 1.0 (2.4 – 6.8)	−0.27	(−0.43, −0.11)	4.4 ± 1.0 (2.7 - 8.5)	4.2 ± 1.0 (2.5 – 6.5)	−0.15	(−0.34, 0.04)	0.60
LDL Cholesterol	<2.5	2.5 ± 0.97 (0.8 – 5.1)	2.2 ± 0.9 (0.5 – 4.1)	−0.25	(−0.40, −0.10)	2.4 ± 0.86 (1.1 - 6.0)	2.2 ± 0.8 (0.9 – 4.2)	−0.15	(−0.32, 0.02)	0.49
HDL Cholesterol	>1.0	1.3 ± 0.34 (0.64 – 2.5)	1.3 ± 0.4 (0.5 – 2.5)	−0.02	(−0.07, 0.03)	1.2 ± 0.37 (0.6 – 2.5)	1.3 ± 0.4 (0.6 – 2.3)	0.03	(−0.01, 0.06)	0.31
Chol./HDL	<4.5	3.7 ± 1.1 (1.5 - 7.6)	3.6 ± 1.3 (1.7 – 9.8)	−0.09	(−0.30, 0.11)	3.8 ± 1.2 (1.8 - 7.3)	3.5 ± 1.2 (1.8 – 7.7)	−0.22	(−0.41, −0.03)	0.29
LDL-HDL	<3.7	2.0 ± 1.0 (0.4 – 5.4)	1.9 ± 1.0 (0.4 – 6.8)	−0.10	(−0.27, 0.06)	2.0 ± 0.9 (0.6 – 4.8)	1.8 ± 0.8 (0.7 – 4.3)	−0.16	(−0.32, -0.01)	0.33
Triglycerides	<1.5	1.7 ± 0.8 (0.5 – 5.4)	1.7 ± 1.0 (0.5 – 5.5)	0.07	(−0.10, 0.24)	1.7 ± 0.9 (0.5 – 5.8)	1.6 ± 0.9 (0.6 – 5.0)	0.09	(−0.08, 0.25)	0.82

^*^ *p*-value is calculated for comparing mean change between groups

At baseline, only 16 % (17/108) usual care arm participants and 23 % (22/98) intervention arm participants had HbA1c > 8 % (>64 mmol/mol), and 12 % (12/108) and 13 % (11/98), respectively, had HbA1c ≥ 8.5 % (>69 mmol/mol). Post hoc analysis showed no evidence of a peer group support effect on changing from baseline to 12 months the proportions of participants with either HbA1c ≥ 8.0 % (≥69 mmol/mol), *p* = 0.49, or HbA1c ≥ 8.5 % (≥69 mmol/mol), *p* = 0.47.

### Behavioural and other secondary outcomes

While intervention participants showed some improvement in self-reported medication adherence after 12 months of peer support, this was not significantly different to usual care participants (OR 2.5 (95 % CI 0.9, 7.0, *p* = 0.09)). With intervention there were greater improvements in the number of days undertaking self-care behaviours compared with usual care (Additional file 1: Table S1) which included increases in the number of days eating >5 serves of fruit and vegetables per day (*p* < 0.01), participating in a specific exercise session (*p* = 0.03) and testing blood sugar (*p* = 0.02). Both study arms showed increased frequency in physical activity more than three times per week, with the difference between them (intervention 15.4 % vs. usual care 9.1 % increase) not statistically significant (*p* = 0.61).

Recipients of peer support also reported a significant improvement in the availability of (*p* = 0.01) and satisfaction with support (*p* < 0.01) from their health care team after 12 months of the intervention, compared to the usual care group at 12 months (Additional file 1: Table S2). No significant improvement was observed in the intervention compared to usual care group with respect to mental health or change of depression category over time (data not shown). Diabetes distress as measured by the brief diabetes distress scale −4 (DDS4) was not significantly improved for those receiving the intervention compared to those receiving usual care (data not shown). In general, levels of distress over the three domains investigated in our participants were not of clinical significance given most mean measures by study arm were below the clinical indicator of moderate psychological distress (<3.0) [33, 43].

While intervention participants did report a slight increase (5 %) in health status over time, these potential improvements were not significant when comparing the weighted EuroQuol EQ5D™ index score between intervention and usual care participants (*p* = 0.34) (data not shown).

### Evaluation of diabetes self-management education

All participants received DSME prior to group allocation. Ninety four percent of participants were “very” or “extremely satisfied” with the education session. Seventy seven percent of the participants reported their diabetes knowledge and understanding had “improved a lot”. Most participants reported that they intended to change their diabetes management after the session with 43 % reporting intention to make changes to their diet, 33 % reported intention to change exercise and more than 19 % reported intention to make changes to blood glucose monitoring. Dietary information was reported as most helpful by 17 % of participants; effects/consequences of diabetes by 17 %, medication by 8 %, and 37 % of participants described all aspects of the DSME as most helpful.

### Evaluation of program implementation

Eleven of the 12 groups initiated and implemented the program. One location (cluster) was allocated to receive the program but insufficient group members were recruited, hence this group joined with another location (Fig. 1). Due to delayed initiation of this combined group the intervention lasted only 8 months during which time this group met on eight occasions. The other 11 groups met at least 12 times over the 12-month period. Regular meeting reports were obtained each month for at least the first 6 months of the intervention from eight of the 11 groups. Additionally, verbal reports of meeting numbers and topics discussed at the meetings were reviewed during the weekly teleconference call between peer supporters and members of the project team. Seven of 11 groups recorded an average attendance of more than 63 % of members at the group meetings, two groups did not provide regular data on attendance and a further two groups reported an average attendance at the group meetings of 35 and 53 % over the 12-month intervention.

Healthy diet, physical activity, goal setting, problem solving and clinical care and medication adherence were regularly discussed during at least three meetings in all groups. Mental health and coping skills were discussed at least once by all groups. In addition to regular phone contact between peer supporters and their group participants, seven of the 11 groups also had regular interaction outside the formal group sessions, including regular walking as a group, Tai Chi class and/or social meetings outside the group meeting. Additional interactions were reported orally during the teleconference by peer supporters and their frequency was group dependent. Organised walks by one group occurred at least once between group meetings during the intervention period. Tai-Chi classes occurred for one group approximately every second month. Social meetings outside the group consisted of activities such as meeting for coffee or opportunistic interaction in community settings such as during shopping. Only one group reported discussing sexual health during their group sessions. Eight of the 11 groups reported visits to their formal group sessions from two or more local health or allied health professionals, including doctors, psychologists, sports physiologists, pharmacists, podiatrists, diabetes educators and dieticians.

## Discussion

In this “real world” trial we evaluated the outcomes and implementation of community based lay peer led support groups on cardiovascular disease risk, biochemical and anthropometric measures and self-management behaviours among participants with T2DM. Even though there were positive changes on some key self-management behaviours, these were not sufficient to reduce cardiovascular disease risk after 12 months. Both the intervention and the usual care groups showed small improvements in most of the biochemical and anthropometric outcomes. There are three explanations for our findings: firstly, peer support as delivered in our program was not effective in improving clinical outcomes such as HbA1c; secondly, program exposure in our intervention was not sufficient to effect change in intervention participants; or thirdly, the study participants recruited for this trial were already quite well controlled and hence, there was an insufficient number of those whose control was poor in order to detect a significant difference in the main study outcomes. Most likely, our study findings resulted from a combination of these three explanations which are discussed below.

There is increasing evidence that peer and professionally delivered behavioural interventions such as peer health coaching and support or individual diabetes education by health professionals may be more beneficial for patients with lower medication adherence and self-management levels [23], or poor glycaemic control, as indicated by HbA1c greater than 8 % [44–46]. However, any improvement in medication adherence in our study, did not translate to improved clinical outcomes such as reduced HbA1c or lipid measures. Furthermore, a post hoc analysis of those study participants with poorer control at baseline found no significant benefit of peer support for these participants, although the number of study participants who were poorly controlled at baseline was quite small.

While the average proportion of participants attending monthly group meetings in this “real world” trial was 63 % or less, several studies have suggested that programs with higher contact and intensity are likely to achieve better clinical outcomes and improvement in diabetes related self-management behaviours [24, 47]. However, half of our study participants already had quite good diabetes control as well as a good level of diabetes knowledge and most had visited their health care provider and had had their HbA1c tested at least once in the six months prior to the beginning of the study [48]. Additionally, all recruited participants received an intensive day of diabetes self-management education as well as a comprehensive education resource manual prior to allocation of clusters which may have contributed to some of the improvements observed within the usual care group [49].

Notwithstanding the lack of significance in clinical outcomes, it is important to note that intervention participants in our trial did report some positive lifestyle changes. Participants reported increased physical activity and fruit and vegetable consumption. And participants also reported increased satisfaction with support from health care providers.

An earlier review of behavioural interventions for lifestyle change undertaken by Oldenburg and Absetz [50] showed that more comprehensive interventions are generally more effective in disease management than single behavior-focused interventions, and the same applies for more intensive and longer interventions. Furthermore, longer term and more sustained follow-up is also critical to enhance maintenance of the changes [50]. Although the intervention program evaluated in this trial certainly incorporated strategies addressing each of the lifestyle and self-care behaviours that are linked to T2DM and its ongoing management, there was considerable variability in the program dose received by study participants across the groups. Therefore, while the program evaluated in this trial was “real world” and it would be feasible for wider implementation, perhaps the dose and intensity was insufficient for achieving significant outcomes, even among those with poorer adherence and control. Moreover, maintaining participant’s involvement and engagement during the intervention (such as regular attendance at group meetings and supporting each other), could be an important determining factor for the successful delivery of diabetes self-management interventions particularly for those whose diabetes is less well controlled.

A key strength of this study was the “real world” nature of the program and its delivery by lay people with diabetes. This is a very important issue for future program scalability because it is probably not feasible for the health care system to provide such programs directly to all people with diabetes or potentially even, those who have poor control and other difficulties with diabetes self-management. At least within the Australian context, non-government organisations such as Diabetes Australia are often relied upon to support the development and ongoing management of these programs. Another strength of our study is the fact that the evaluation design also allowed for a thorough evaluation of its impact and its future potential for scalability.

Eleven of the 12 proposed community based peer groups were initiated and remained active for at least 12 months. With several groups continuing to meet after the final measurements were collected. Thus, we have demonstrated that it is possible to develop and implement a “real world” community based peer support program for people with T2DM which enables members to make at least some changes in diabetes self-management behaviours such as physical activity and diet. We also observed some significant improvement in the satisfaction and perception of support from diabetes health care team, which may assist in improving the collaborative relationship between patient and health care team and thus, also improve medication adherence in the future.

As demonstrated by our relatively low crude response rate (6.9 %) recruitment via the national register for people with diabetes in Australia, while feasible and cost effective, may not be appropriate for reaching those individuals with poor diabetes control and also experiencing other difficulties.

The major study limitation was the low response rate and the fact that almost half of those individuals recruited already had reasonably good glycaemic control. This is despite the fact that our recruitment method of sending a direct mail invitation to registrants of a National Diabetes registry (such as might be the method used in a “real world” community based program) is cost effective and the crude response rate (6.9 %) from this method is similar to that reported in previous studies [51].

Unfortunately, two of the intervention groups did not provide regular data on meeting details, so this made it difficult to calculate an accurate and reliable measure of program exposure for all groups and all participants. However, program exposure was quite modest in some of the groups and likely to be of insufficient intensity for those experiencing difficulties with diabetes control and related problems.

Several studies in the global Peers for Progress program whose participants included those from disadvantaged communities with poorer diabetes control report benefits of peer support or health coaching directly on HbA1c [8, 52] or on cardiovascular risk factors [53, 54]. Interestingly those projects conducted in populations with better access to either privately or publicly funded health care did not appear to benefit significantly with respect to clinical health outcomes from additional peer support [11, 15, 22].

Despite these various findings and our own results, peer support programs have been shown to be feasible and acceptable at the community level and probably provide some important benefits and outcomes, particularly for those “hard to reach” patient groups [25]. However, important questions still remain about how to better target those subgroups that might derive greater benefit from peer support, such as those who have higher HbA1c levels, those who have poor medication adherence and those who are experiencing other difficulties with diabetes self-management and living with diabetes.

## Conclusions

In summary, we report here on the real world implementation of a community-based, lay led peer support program which aimed to train and support lay people to provide structured support and help to people with diabetes in order to improve diabetes self-management. Although there were some modest benefits in terms of healthy behaviors and participants’ reports of satisfaction with their care, it did not improve clinical outcomes for the majority of participants. Future research needs to focus more on how this kind of program and delivery model could be used to provide more structure support and help to those individuals with diabetes who have poor diabetes control and who are experiencing other significant difficulties living with diabetes.

## Additional file

Additional file 1: Table S1. Diabetes Self-care behaviours: Changes over 12 months between intervention and usual care groups in Diabetes Self-care behaviours. This table details the mean baseline and 12 month measures of diabetes self-care behaviour [35, 55] for the intervention and usual care groups and compares the change overtime between the intervention and usual care group. **Table S2.** MDS – support and satisfaction of support friends/ health care team: Changes over 12 months between intervention and usual care groups in perception of support received. This table details the mean baseline and 12 month measures of perceived support from family and health care team [36] in the intervention and usual care groups and compares the change overtime between the intervention and usual care group. (DOCX 23 kb)

## Abbreviations

BGL: Blood glucose level
BMI: Body mass index
CHD: Coronary heart disease
CONSORT: Consolidated Standards of Reporting Trials
CVD: Cardiovascular disease
DBP: Diastolic blood pressure
DSME: Diabetes self-management education
HbA1c: Glycated haemoglobin
IFCC: International Federation of Clinical Chemistry
LGA: Local Government Authority
NDSS: National Diabetes Services Scheme
SBP: Systolic blood pressure
T2DM: Type 2 diabetes mellitus
UKPDS: United Kingdom Prospective Diabetes Study
WHO STEPS: World Health Organization STEPwise approach to Surveillance

## References

[1] Vos T, Barber RM, Bell B, Bertozzi-Villa A, Biryukov S, Bolliger I, Charlson F, Davis A, Degenhardt L, Dicker D et al: Global, regional, and national incidence, prevalence, and years lived with disability for 301 acute and chronic diseases and injuries in 188 countries, 1990–2013: a systematic analysis for the Global Burden of Disease Study 2013. The Lancet. 2015;386(9995):743-800. 10.1016/S0140-6736(15)60692-4 PMC4561509 26063472

[2] Campbell PT, Newton CC, Patel AV, Jacobs EJ, Gapstur SM (2012). Diabetes and cause-specific mortality in a prospective cohort of one million U.S. adults. Diabetes Care.

[3] Australian Institute of Health and Welfare, Australian Institute of Health and Welfare (2014). Cardiovascular disease, diabetes and chronic kidney disease— Australian facts: mortality. Cardiovascular, diabetes and chronic kidney disease series no 1.

[4] Adler AI, Stratton IM, Neil HA, Yudkin JS, Matthews DR, Cull CA, Wright AD, Turner RC, Holman RR (2000). Association of systolic blood pressure with macrovascular and microvascular complications of type 2 diabetes (UKPDS 36): prospective observational study. BMJ.

[5] Stratton IM, Adler AI, Neil HA, Matthews DR, Manley SE, Cull CA, Hadden D, Turner RC, Holman RR (2000). Association of glycaemia with macrovascular and microvascular complications of type 2 diabetes (UKPDS 35): prospective observational study. BMJ.

[6] Fisher EB, Boothroyd RI, Coufal MM, Baumann LC, Mbanya JC, Rotheram-Borus MJ, Sanguanprasit B, Tanasugarn C (2012). Peer support for self-management of diabetes improved outcomes in international settings. Health Aff.

[7] Heisler M, Vijan S, Makki F, Piette JD (2010). Diabetes control with reciprocal peer support versus nurse care management: a randomized trial. Ann Intern Med.

[8] Thom DH, Ghorob A, Hessler D, De Vore D, Chen E, Bodenheimer TA (2013). Impact of peer health coaching on glycemic control in low-income patients with diabetes: a randomized controlled trial. Ann Fam Med.

[9] Perry HB, Zulliger R, Rogers MM (2014). Community health workers in low-, middle-, and high-income countries: an overview of their history, recent evolution, and current effectiveness. Annu Rev Public Health.

[10] Fisher EB, Coufal MM, Parada H, Robinette JB, Tang PY, Urlaub DM, Castillo C, Guzman-Corrales LM, Hino S, Hunter J (2014). Peer support in health care and prevention: cultural, organizational, and dissemination issues. Annu Rev Public Health.

[11] Chan JC, Sui Y, Oldenburg B, Zhang Y, Chung HH, Goggins W, Au S, Brown N, Ozaki R, Wong RY (2014). Effects of telephone-based peer support in patients with type 2 diabetes mellitus receiving integrated care: a randomized clinical trial. JAMA Intern Med.

[12] Cherrington A, Martin MY, Hayes M, Halanych JH, Wright MA, Appel SJ, Andreae SJ, Safford M (2012). Intervention mapping as a guide for the development of a diabetes peer support intervention in rural Alabama. Prev Chronic Dis.

[13] Dang TTN, Deoisres W, Keeratiyutawong P, Baumann LC (2013). Effectiveness of a Diabetes Self Management support intervention in Vietnamese adults with Type 2 Diabetes. J Sci Technol Humanit.

[14] Rotheram-Borus MJ, Tomlinson M, Gwegwe M, Comulada WS, Kaufman N, Keim M (2012). Diabetes buddies: peer support through a mobile phone buddy system. Diabetes Educ.

[15] Simmons D, Prevost AT, Bunn C, Holman D, Parker RA, Cohn S, Donald S, Paddison CA, Ward C, Robins P (2015). Impact of community based peer support in type 2 diabetes: a cluster randomised controlled trial of individual and/or group approaches. PLoS One.

[16] Smith SM, Paul G, Kelly A, Whitford DL, O’Shea E, O’Dowd T (2011). Peer support for patients with type 2 diabetes: cluster randomised controlled trial. BMJ.

[17] Tang TS, Nwankwo R, Whiten Y, Oney C (2014). Outcomes of a church-based diabetes prevention program delivered by peers: a feasibility study. Diabetes Educ.

[18] Baumann LC, Frederick N, Betty N, Jospehine E, Agatha N (2015). A demonstration of peer support for ugandan adults with type 2 diabetes. Int J Behav Med.

[19] Lorig K, Ritter PL, Villa F, Piette JD (2008). Spanish diabetes self-management with and without automated telephone reinforcement: two randomized trials. Diabetes Care.

[20] Tang TS, Funnell M, Sinco B, Piatt G, Palmisano G, Spencer MS, Kieffer EC, Heisler M (2014). Comparative effectiveness of peer leaders and community health workers in diabetes self-management support: results of a randomized controlled trial. Diabetes Care.

[21] Piette J, Resnicow K, Choi H, Heisler M: A diabetes peer support intervention that improved glycemic control: mediators and moderators of intervention effectiveness. Chronic Illn. 2013;9(4):258-267. 10.1177/1742395313476522 PMC3830685 23585636

[22] Knox L, Huff J, Graham D, Henry M, Bracho A, Henderson C, Emsermann C (2015). What peer mentoring adds to already good patient care: implementing the carpeta roja peer mentoring program in a well-resourced health care system. Ann Fam Med.

[23] Moskowitz D, Thom DH, Hessler D, Ghorob A, Bodenheimer T (2013). Peer coaching to improve diabetes self-management: which patients benefit most?. J Gen Intern Med.

[24] Qi L, Liu Q, Qi X, Wu N, Tang W, Xiong H (2015). Effectiveness of peer support for improving glycaemic control in patients with type 2 diabetes: a meta-analysis of randomized controlled trials. BMC Public Health.

[25] Fisher EB, Ballesteros J, Bhushan N, Coufal MM, Kowitt SD, McDonough AM, Parada H, Robinette JB, Sokol RL, Tang PY (2015). Key features of peer support in chronic disease prevention and management. Health Aff (Millwood).

[26] Boothroyd RI, Fisher EB (2010). Peers for progress: promoting peer support for health around the world. Fam Pract.

[27] Fisher EB, Earp JA, Maman S, Zolotor A (2010). Cross-cultural and international adaptation of peer support for diabetes management. Fam Pract.

[28] Riddell MA, Renwick C, Wolfe R, Colgan S, Dunbar JA, Hagger V, Absetz P, Oldenburg B, on behalf of the Australasian Peers for Progress Diabetes Project Investigators (2012). Cluster randomized controlled trial of a peer support program for people with diabetes: Study protocol for the Australasian Peers for Progress study. BMC Public Health.

[29] Campbell MK, Piaggio G, Elbourne DR, Altman DG (2012). Consort 2010 statement: extension to cluster randomised trials. BMJ.

[30] National Diabetes Sevices Scheme, [https://www.ndss.com.au/].

[31] World Health Organization, Department of Chronic Diseases and Health Promotion. Section 3: Guide to Physical Measurements (Step 2). In: Part 3: Training and Practical Guides, WHO STEPS Surveillance Manual - Updated: 13 June 2008. Geneva: World Health Organization; 2008. [http://www.who.int/chp/steps/Part3.pdf?ua=1].

[32] Rabin R, de Charro F (2001). EQ-5D: a measure of health status from the EuroQol Group. Ann Med.

[33] Fisher L, Glasgow RE, Mullan JT, Skaff MM, Polonsky WH (2008). Development of a brief diabetes distress screening instrument. Ann Fam Med.

[34] Kroenke K, Spitzer RL, Williams JB (2001). The PHQ-9: validity of a brief depression severity measure. J Gen Intern Med.

[35] Toobert DJ, Hampson SE, Glasgow RE (2000). The summary of diabetes self-care activities measure: results from 7 studies and a revised scale. Diabetes Care.

[36] Tang TS, Brown MB, Funnell MM, Anderson RM (2008). Social support, quality of life, and self-care behaviors among African Americans with type 2 diabetes. Diabetes Educ.

[37] Manual General Practice Assessment Questionnaire (GPAQ) version 2.1 [http://www.gpaq.info/GPAQmanualV2_1.pdf]

[38] Jones GR, Barker G, Goodall I, Schneider HG, Shephard MD, Twigg SM (2011). Change of HbA1c reporting to the new SI units. Med J Aust.

[39] Stevens RJ, Kothari V, Adler AI, Stratton IM (2001). The UKPDS risk engine: a model for the risk of coronary heart disease in Type II diabetes (UKPDS 56). Clin Sci (Lond).

[40] Vargas RB, Mangione CM, Asch S, Keesey J, Rosen M, Schonlau M, Keeler EB (2007). Can a chronic care model collaborative reduce heart disease risk in patients with diabetes?. J Gen Intern Med.

[41] Spitzer RL, Kroenke K, Williams JB (1999). Validation and utility of a self-report version of PRIME-MD: the PHQ primary care study. Primary Care Evaluation of Mental Disorders. Patient Health Questionnaire. JAMA.

[42] Morisky DE, Green LW, Levine DM (1986). Concurrent and predictive validity of a self-reported measure of medication adherence. Med Care.

[43] Polonsky WH, Fisher L, Earles J, Dudl RJ, Lees J, Mullan J, Jackson RA (2005). Assessing psychosocial distress in diabetes: development of the diabetes distress scale. Diabetes Care.

[44] Duke SA, Colagiuri S, Colagiuri R (2009). Individual patient education for people with type 2 diabetes mellitus. Cochrane Database Syst Rev.

[45] Lorig K, Ritter PL, Ory MG, Whitelaw N (2013). Effectiveness of a generic chronic disease self-management program for people with type 2 diabetes: a translation study. Diabetes Educ.

[46] Lorig K, Ritter PL, Villa FJ, Armas J (2009). Community-based peer-led diabetes self-management: a randomized trial. Diabetes Educ.

[47] Barr EL, Zimmet PZ, Welborn TA, Jolley D, Magliano DJ, Dunstan DW, Cameron AJ, Dwyer T, Taylor HR, Tonkin AM (2007). Risk of cardiovascular and all-cause mortality in individuals with diabetes mellitus, impaired fasting glucose, and impaired glucose tolerance: the Australian Diabetes, Obesity, and Lifestyle Study (AusDiab). Circulation.

[48] Rawal LB, Wolfe R, Joyce C, Riddell M, Dunbar JA, Li H, Oldenburg B: Utilisation of general practitioner services and achievement of guideline targets by people with diabetes who joined a peer-support program in Victoria, Australia. Aust J Prim Health. 2014;21(2):205-213. 10.1071/PY13178 24618400

[49] Funnell MM, Brown TL, Childs BP, Haas LB, Hosey GM, Jensen B, Maryniuk M, Peyrot M, Piette JD, Reader D (2012). National standards for diabetes self-management education. Diabetes Care.

[50] Oldenburg B, Absetz P, Chan CKY, Steptoe A, Freedland K, Jenning JR, Llabre M, Manuck S, Susman E (2010). Behavioural interventions for prevention and management of chronic disease. Handbook of behavioural medicine: methods and applications. edn.

[51] Sinclair M, O’Toole J, Malawaraarachchi M, Leder K (2012). Comparison of response rates and cost-effectiveness for a community-based survey: postal, internet and telephone modes with generic or personalised recruitment approaches. BMC Med Res Methodol.

[52] Ayala GX, Ibarra L, Cherrington AL, Parada H, Horton L, Ji M, Elder JP (2015). Puentes hacia una mejor vida (bridges to a better life): outcome of a diabetes control peer support intervention. Ann Fam Med.

[53] Safford MM, Andreae S, Cherrington AL, Martin MY, Halanych J, Lewis M, Patel A, Johnson E, Clark D, Gamboa C (2015). Peer coaches to improve diabetes outcomes in rural Alabama: a cluster randomized trial. Ann Fam Med.

[54] Tang TS, Funnell MM, Sinco B, Spencer MS, Heisler M (2015). Peer-Led, Empowerment-Based Approach to Self-Management Efforts in Diabetes (PLEASED): a randomized controlled trial in an African American Community. Ann Fam Med.

[55] Macera CA, Ham SA, Jones DA, Kimsey CD, Ainsworth BE, Neff LJ (2001). Limitations on the use of a single screening question to measure sedentary behavior. Am J Public Health.

