# Supplementary material for: Cardiovascular risk outcome and program evaluation of a cluster randomised controlled trial of a community-based, lay peer led program for people with diabetes
Source: BMC Public Health. 2016 Aug 24;16(1):864. doi: 10.1186/s12889-016-3538-3 (PMC4995663; doi:10.1186/s12889-016-3538-3)
Supplement: Additional file 1: Table S1. — Diabetes Self-care behaviours: Changes over 12 months between intervention and usual care groups in Diabetes Self-care behaviours. This table details the mean baseline and 12 month measures of diabetes self-care behaviour [35, 55] for the intervention and usual care groups and compares the change overtime between the intervention and usual care group. Table S2. MDS – support and satisfaction of support friends/ health care team: Changes over 12 months between intervention and usual care groups in perception of support received. This table details the mean baseline and 12 month measures of perceived support from family and health care team [36] in the intervention and usual care groups and compares the change overtime between the intervention and usual care group. (DOCX 23 kb) [file 12889_2016_3538_MOESM1_ESM.docx]

**Additional file 1: Table S1: Diabetes Self-care behaviours: Changes over 12 months between intervention and usual care groups in Diabetes Self-care behaviours**

|  | Usual Care | | | Intervention | | | |  |
| --- | --- | --- | --- | --- | --- | --- | --- | --- |
|  |  | baseline | 12mth |  | baseline | | 12mth | P value |
| How many days? | n | mean (SD) | mean (SD) | n | | mean (SD) | mean (SD) | Inter group^1^ |
| >5 servings of fruit/Veg | 97 | 4.6(2.0) | 4.4(2.1) | 88 | | 4.7(2.1) | 5.3(1.9) | **<0.01** |
| High fat foods | 98 | 2.7(2.0) | 2.1(1.6) | 85 | | 2.1(1.4) | 2.1(1.6) | 0.49 |
| Specific Exercise session | 98 | 3.2(2.5) | 3.1(2.4) | 87 | | 2.9(2.5) | 3.7(2.6) | **0.03** |
| 30 mins total physical activity | 94 | 3.2(2.4) | 3(2.4) | 87 | | 2.9(2.5) | 3.4(2.6) | 0.13 |
| Test blood sugar | 94 | 4.6(2.7) | 4.3(2.8) | 91 | | 4.5(2.6) | 4.9(2.6) | **0.02** |
| # Times test BGL/day | 94 | 2.0(1.7) | 1.6(1.4) | 90 | | 1.8(1.6) | 1.9(1.5) | 0.06 |
| Check feet | 97 | 3.2(2.7) | 3.6(2.8) | 90 | | 2.9(2.6) | 3.6(2.9) | 0.74 |

^1^ Intergroup p value is the comparison of changes between intervention and usual care

[1, 2]

**Table S2: MDS – support and satisfaction of support friends/ health care team: Changes over 12 months between intervention and usual care groups in perception of support received**

|  | Usual Care | | | Intervention | | |  |
| --- | --- | --- | --- | --- | --- | --- | --- |
|  |  | baseline | 12mth |  | baseline | 12mth | P |
|  | n | mean (SD) | mean (SD) | n | mean (SD) | mean (SD) | Inter-group |
| How much support from family and friends | 84 | 3.0(1.5) | 3.1(1.5) | 76 | 3.5(1.4) | 3.7(1.4) | 0.16 |
| Satisfaction with support from family and friends | 78 | 3.6(1.4) | 3.7(1.4) | 69 | 4.0(1.0) | 4.3(0.9) | 0.09 |
| How much support from health care team | 77 | 3.1(1.4) | 3.3(1.5) | 67 | 3.6(1.3) | 4.0(1.1) | **0.01** |
| Satisfaction with support from health care team | 72 | 3.7(1.4) | 3.8(1.3) | 69 | 3.9(1.1) | 4.3(0.9) | **<0.01** |

Ref [3]

**References**

1. Macera CA, Ham SA, Jones DA, Kimsey CD, Ainsworth BE, Neff LJ: **Limitations on the use of a single screening question to measure sedentary behavior**. *Am J Public Health* 2001, **91**(12):2010-2012.

2. Toobert DJ, Hampson SE, Glasgow RE: **The summary of diabetes self-care activities measure: results from 7 studies and a revised scale**. *Diabetes Care* 2000, **23**(7):943-950.

3. Tang TS, Brown MB, Funnell MM, Anderson RM: **Social support, quality of life, and self-care behaviors among African Americans with type 2 diabetes**. *The Diabetes educator* 2008, **34**(2):266-276.
